# Supplementary material for: P-TRAP: a Panicle Trait Phenotyping tool
Source: BMC Plant Biol. 2013 Aug 29;13:122. doi: 10.1186/1471-2229-13-122 (PMC3848748; doi:10.1186/1471-2229-13-122)
Supplement: Additional file 7 — P-TRAP source code. The Netbeans project that contains the source code of the application. [file 1471-2229-13-122-S7.zip › javadoc.zip!/javadoc/org-bioalg-skeletonprocessor/org/bioalg/skeletonprocessor/SkeletonProcessor.html]

SkeletonProcessor (SkeletonProcessor)


JavaScript is disabled on your browser.


- Package
- Class
- Tree
- Deprecated
- Index
- Help

*org.bioalg.skeletonprocessor 1.0*

- Prev Class
- Next Class

- Frames
- No Frames

- All Classes

- Summary:
- Nested |
- Field |
- Constr |
- Method

- Detail:
- Field |
- Constr |
- Method


org.bioalg.skeletonprocessor

## Class SkeletonProcessor

- java.lang.Object
- - org.bioalg.skeletonprocessor.SkeletonProcessor

- ---

    

  ```
  public class SkeletonProcessor
  extends java.lang.Object
  ```

  This is the main class. It processes the skeleton based on graph theory.

  Author:
  :   faroq

- - ### Constructor Summary

    Constructors

    Constructor and Description

    `SkeletonProcessor()`
  - ### Method Summary

    Methods

    | Modifier and Type | Method and Description |
    | --- | --- |
    | `void` | `buildGraph(java.awt.Point currentPoint, java.awt.Point lastPoint)` Recursively build a graph from the skeleton points |
    | `void` | `follow(java.awt.Point currentPoint, Skeleton mainskeleton, Skeleton newSkeleton)` Follow the skeleton recursively starting at a given point. |
    | `java.util.ArrayList<Skeleton>` | `getAllSkeletons()` Return all skeletons from an image or a super skeleton. |
    | `java.util.ArrayList<Skeleton>` | `getAllSkeletons(org.bioalg.imageprocessor.AImage skeletonImage, int minSkeletonSize)` Find all skeletons in a given skeleton image. |
    | `org.bioalg.graphprocessor.Graph` | `getGraph()` |
    | `Skeleton` | `getSkeleton()` |
    | `org.bioalg.imageprocessor.AImage` | `getSkeletonimg()` |
    | `java.util.ArrayList<java.awt.Point>` | `getVisited()` |
    | `int` | `removeSpikes(double threshold)` Removes spikelets from the skeleton given a threshold |
    | `int` | `removeTransitionVertices()` Removes any transition points |
    | `void` | `restVisited()` |
    | `void` | `setGraph(org.bioalg.graphprocessor.Graph graph)` |
    | `void` | `setSkeleton(org.bioalg.imageprocessor.AImage img)` Set the skeleton from an image As different skeletons may exist in the image, this skeleton can be represented as main skeleton. |
    | `void` | `setSkeleton(Skeleton skeleton)` |
    | `void` | `setSkeletonimg(org.bioalg.imageprocessor.AImage skeletonimg)` |
    | `void` | `setVisited(java.util.ArrayList<java.awt.Point> visited)` |
    | `void` | `Unify(int threshold)` Remove the corwoded nodes in the graph given a threshold |

    - ### Methods inherited from class java.lang.Object

      `clone, equals, finalize, getClass, hashCode, notify, notifyAll, toString, wait, wait, wait`

- - ### Constructor Detail


    - #### SkeletonProcessor

      ```
      public SkeletonProcessor()
      ```
  - ### Method Detail


    - #### restVisited

      ```
      public void restVisited()
      ```


    - #### getGraph

      ```
      public org.bioalg.graphprocessor.Graph getGraph()
      ```


    - #### setGraph

      ```
      public void setGraph(org.bioalg.graphprocessor.Graph graph)
      ```


    - #### getSkeleton

      ```
      public Skeleton getSkeleton()
      ```


    - #### setSkeleton

      ```
      public void setSkeleton(org.bioalg.imageprocessor.AImage img)
      ```

      Set the skeleton from an image
      As different skeletons may exist in the image,
      this skeleton can be represented as main skeleton.

      Parameters:
      :   `img` - the input image


    - #### buildGraph

      ```
      public void buildGraph(java.awt.Point currentPoint,
                    java.awt.Point lastPoint)
      ```

      Recursively build a graph from the skeleton points

      Parameters:
      :   `currentPoint` -
      :   `lastPoint` -


    - #### follow

      ```
      public void follow(java.awt.Point currentPoint,
                Skeleton mainskeleton,
                Skeleton newSkeleton)
      ```

      Follow the skeleton recursively starting at a given point.

      Parameters:
      :   `currentPoint` -
      :   `lastPoint` -
      :   `newSkeleton` - the new created skeleton.


    - #### getAllSkeletons

      ```
      public java.util.ArrayList<Skeleton> getAllSkeletons(org.bioalg.imageprocessor.AImage skeletonImage,
                                                  int minSkeletonSize)
      ```

      Find all skeletons in a given skeleton image.

      Parameters:
      :   `skeletonImage` - the input maximumSkeleton image

      Returns:
      :   a list of skeletons


    - #### getAllSkeletons

      ```
      public java.util.ArrayList<Skeleton> getAllSkeletons()
      ```

      Return all skeletons from an image or a super skeleton.

      Returns:


    - #### setSkeleton

      ```
      public void setSkeleton(Skeleton skeleton)
      ```


    - #### getSkeletonimg

      ```
      public org.bioalg.imageprocessor.AImage getSkeletonimg()
      ```


    - #### setSkeletonimg

      ```
      public void setSkeletonimg(org.bioalg.imageprocessor.AImage skeletonimg)
      ```


    - #### getVisited

      ```
      public java.util.ArrayList<java.awt.Point> getVisited()
      ```


    - #### setVisited

      ```
      public void setVisited(java.util.ArrayList<java.awt.Point> visited)
      ```


    - #### Unify

      ```
      public void Unify(int threshold)
      ```

      Remove the corwoded nodes in the graph given a threshold


    - #### removeTransitionVertices

      ```
      public int removeTransitionVertices()
      ```

      Removes any transition points

      Returns:
      :   number of removed transition points removed


    - #### removeSpikes

      ```
      public int removeSpikes(double threshold)
      ```

      Removes spikelets from the skeleton given a threshold

      Parameters:
      :   `threshold` -

      Returns:
      :   number of removed spikes


- Package
- Class
- Tree
- Deprecated
- Index
- Help

*org.bioalg.skeletonprocessor 1.0*

- Prev Class
- Next Class

- Frames
- No Frames

- All Classes

- Summary:
- Nested |
- Field |
- Constr |
- Method

- Detail:
- Field |
- Constr |
- Method
